# Barriers and facilitators for disease registry systems: a mixed-method study

**DOI:** 10.1186/s12911-022-01840-7

**Published:** 2022-04-11

**Authors:** Mina Lazem, Abbas Sheikhtaheri

**Affiliations:** Department of Health Information Management, School of Health Management and Information Sciences, Iran University of Medical Sciences, Tehran, Iran

**Keywords:** Disease registry systems, Barrier, Facilitator, Iran

## Abstract

**Background:**

A Disease Registry System (DRS) is a system that collects standard data on a specific disease with an organized method for specific purposes in a population. Barriers and facilitators for DRSs are different according to the health system of each country, and identifying these factors is necessary to improve DRSs, so the purpose of this study was to identify and prioritize these factors.

**Methods:**

First, by conducting 13 interviews with DRS specialists, barriers and facilitators for DRSs were identified and then, a questionnaire was developed to prioritize these factors. Then, 15 experts answered the questionnaires. We prioritized these factors based on the mean of scores in four levels including first priority (3.76–5), second priority (2.51–3.75), third priority (1.26–2.50), and the fourth priority (1–1.25).

**Results:**

At first, 139 unique codes (63 barriers and 76 facilitators) were extracted from the interviews. We classified barriers into 9 themes, including management problems (24 codes), data collection-related problems (8 codes), poor cooperation/coordination (7 codes), technological problems and lack of motivation/interest (6 codes for each), threats to ethics/data security/confidentiality (5 codes), data quality-related problems (3 codes), limited patients’ participation and lack of or non-use of standards (2 codes for each). We also classified facilitators into 9 themes including management facilitators (36 codes), improving data quality (8 codes), proper data collection and observing ethics/data security/confidentiality (7 codes for each), appropriate technology (6 codes), increasing patients’ participation, increasing motivation/interest, improving cooperation/coordination, and the use of standards (3 codes for each). The first three ranked barriers based on mean scores included poor stakeholder cooperation/coordination (4.30), lack of standards (4.26), and data quality-related problems (4.06). The first three ranked facilitators included improving data quality (4.54), increasing motivation/interest (4.48), and observing ethics/data security/confidentiality (4.36).

**Conclusion:**

Stakeholders’ coordination, proper data management, standardization and observing ethics, security/confidentiality are the most important areas for planning and investment that managers must consider for the continuation and success of DRSs.

**Supplementary Information:**

The online version contains supplementary material available at 10.1186/s12911-022-01840-7.

## Background

A Disease Registry System (DRS) is a system with one or more specific purposes in which standard data about a pre-determined population of patients with the same disease/condition/outcome are continuously collected, analyzed, and reported [1, 2]. These systems are important for various clinical purposes and health research. DRSs may be clinical-based that are used for the evaluation of healthcare, or population-based for estimation of incidence and prevalence [3]. These systems can generally be used to monitor the quality of care and provide relevant feedback, description of treatment plans [4], observation of the natural course of diseases, monitoring of clinical safety, assessment of disease burden, health outcomes, and costs of the diseases [1].

Properly designed and implemented DRSs can provide a true picture of clinical practice, patient outcomes, safety, and comparative effectiveness. There are various stakeholders in the public and private sectors (such as policymakers, hospitals, healthcare centers, and health research centers) that can use DRSs to analyze and interpret patients' data for research purposes or health policymaking [5, 6].

Different stakeholders understand the value of DRSs in different ways and may benefit from it [7]. For example, for a physician, DRSs can rapidly collect data on disease manifestations and their outcomes in a large number of patients and provide a true picture of the disease, current treatments, and outcomes. For a health care organization such as a hospital, a DRS may provide data that is useful for assessing physicians' management of a disease in accordance with evidence-based guidelines. For a drug manufacturer, a DRS-based study may demonstrate the effectiveness of a drug product in the real world. On the other hand, various studies and clinical trials have important roles in assessing patient outcomes. In this regard, DRSs comprehensively collect data and produce results that may be generalizable to a wide range of populations. Therefore, researchers can also use DRSs for various research purposes [5, 7].

The DRSs face various barriers and facilitators [8–11]. The lack of interoperability of registries with other systems [12–14], insufficient financial resources, the lack of staff, and poor data quality [15] are among the barriers. On the other hand, providing resources and adequate training [16], user-friendly software [10], observing patients’ confidentiality, improving data quality [15], and using specific guidelines [17–19] can be considered facilitators.

The Iranian health system is planned and managed by the Ministry of Health and Medical Education (MOHME). Provincial medical universities are responsible for providing health services and medical education in each province. Diagnostic and therapeutic measures are performed by university hospitals in each region (province). These universities are also responsible for evaluating private hospitals in each region. The MOHME is responsible for setting up national DRSs to be implemented in all different provinces. Some researchers in collaboration with the MOHME are also able to develop national DRSs. Depending on the needs, regional DRSs are also set up in hospitals or clinical research centers in different provinces. Specialists and researchers are mainly major users of these systems who use the registered data in research and quality improvement of patient services [20]. MOHME also uses national DRSs for policy-making. Since 2014, the MOHME has supported DRSs; therefore, many disease registries have been developed at the regional or national levels using financial support from the universities and the MOHME. This program is still ongoing [20]. Although there are many studies regarding barriers and facilitators for DRSs in the world, there is little information in this regard in Iran.

Considering the importance of DRSs [21] and also the variety of barriers and facilitators for these systems based on the health system of each country [22], it seems that the disease registry officials should try to identify and prioritize these factors to play an effective role to improve disease registry programs. Identifying these factors may also be important for other developing countries or individual disease registry systems to improve these programs. Therefore, we aimed to identify and prioritize barriers and facilitators for DRSs in Iran.

## Methods

This mixed-method study was conducted in two phases. Barriers and facilitators were identified through a qualitative (content analysis) study and then prioritized using a quantitative method.

### Phase 1: Identifying barriers and facilitators for disease registry systems

#### Participants

We approached experts of DRSs in medical universities and the MOHME as candidates for the interview due to their knowledge and experience. To achieve maximum variety in perspectives, sampling was done purposefully with specific criteria. The selection criterion was at least two years of work experience in DRSs. Participants with a variety of backgrounds from different cities and various registries were invited. Experts were invited from different disease registry systems with different roles in these systems including registrars, researchers, executive directors, quality experts, administrators, or supervisors.

We continued sampling and conducting interviews until data saturation. In the process of achieving data saturation, the majority of codes were identified in the first 11 interviews, followed by a decrease in the number of codes identified in subsequent interviews, which indicates data saturation. Two additional experts were also interviewed to ensure data saturation. Finally, 13 experts were interviewed and three refused to be interviewed due to their busy schedules.

#### Data collection

There are some literature and frameworks regarding barriers and facilitators for disease registries. Based on a review of this literature and frameworks [9, 14, 15, 18, 23–25], we developed a semi-structured interview guide (Additional file 1) with 8 main questions. According to the interview guide, we asked questions about experts’ experiences with DRSs, the barriers and problems they have encountered in the implementation, setting up, running, continuity, and sustainability of DRSs, and subsequently the proposed solutions and facilitators. Therefore, in this study, we considered all the steps from design and implementation to the use of DRSs. Two experts read the interview guide and confirmed the transparency of the questions. The characteristics of the participants were also recorded at the end of each interview (Tables 1, 2).

**Table 1:** Demographic, educational and professional characteristics of the participants in phase 1

Expert	Age	Gender	Field of study	Type of degree	Work experience in DRSs (year)	Activity in DRSs	Geographical scope of registry	Type of registry
P1	42	Female	General Practitioner and Healthcare Services Management	Clinical and non-clinical	10	Researcher and executive director	National	Clinical/population based
P2	28	Female	Nutrition	Clinical	3	Registrar and executive director	National	Clinical/population based
P3	52	Female	Medical Physiology	Clinical	6	Supervisor and evaluator	National/regional	Research/clinical/population-based
P4	42	Male	General Practitioner and Epidemiology	Clinical and non-clinical	17	Executive director	National/regional	Research/ clinical/population-based
P5	33	Female	Health Information Management	Non-clinical	5	Executive director	National	Clinical
P6	41	Female	Endocrinology	Clinical	2	Registrar and data quality expert	National	Research/clinical –based
P7	30	Female	Optometry	Clinical	4	Executive director	National	Research/clinical/population-based
P8	49	Male	Epidemiology	Non-clinical	16	Supervisor and evaluator	National/regional	Research/clinical/population-based
P9	43	Male	General Practitioner and Epidemiology	Clinical and non-clinical	13	Executive director	National	Clinical/population based
P10	51	Female	Maternal and Child Health	Clinical	13	Executive director	Regional	Research/clinical based
P11	60	Female	Medical Physiology	Clinical	2	Executive director	Regional	Research/clinical based
P12	39	Male	Health Information Management	Non-clinical	12	Executive director	National	Clinical
P13	57	Male	General Surgery	Clinical	7	Administrator	National/regional	Research/clinical/population-based

**Table 2:** Distribution of the participants in phase 1

Characteristics	Phase 1 participants. (n = 13)	
	Number (percent)	
Age	< 40 years	9 (69.2)
	≥ 40 years	4 (30.7)
Gender	Female	8 (61.5)
	Male	5 (38.4)
Duration of work in the field of DRS (years)	< 5	4 (30.7)
	Equal to and more than 5	11 (84.6)
Activity in the field of DRS (Each participant may have more than one item)	Supervisor	2 (15.3)
	Evaluator	1 (7.6)
	Principal investigator	0
	Executive director	9 (69.2)
	Administrator	1 (7.6)
	Registrar	2 (15.3)
	Researcher	1 (7.6)
Field of study (each participant may have more than one item)	Endocrinology	1 (7.6)
	General surgery	1 (7.6)
	Healthcare services management	1 (7.6)
	Medical physiology	2 (15.3)
	Epidemiology	2 (15.3)
	Health information management	2 (15.3)
	Optometry	1 (7.6)
	Maternal and child health	1 (7.6)
	General practitioner	3 (23)
	Nutrition	1 (7.6)
Type of degree	Clinical	7 (53.8)
	Non-clinical	3 (23)
	Both clinical and non-clinical	3 (23)
Geographical scope of registry	National	9 (69.2)
	Regional	4 (30.7)
Type of registry	Clinical-based	2 (15.3)
	Clinical/population-based	3 (23)
	Research/clinical-based	3 (23)
	Research/clinical/population-based	5 (38.4)

To achieve greater clarity and depth of the concepts, the first two interviews were conducted as a pilot, and based on the analysis, questions were revised. These two interviews were considered in the final analysis. Most interviews were conducted face to face at the participants’ workplace and four were online. All interviews were conducted by a trained researcher (M.L. who is a Ph.D. candidate in health information management with experience in DRSs). She audio recorded the interviews and took notes. In seven cases, interviews were completed in two or three sessions. The duration of each interview was between 25 and 50 min.

We conducted several measures to increase rigour in data collection. For this purpose, three out of four rigour criteria including (1) validity, (2) reliability and dependability, (3) credibility, and (4) transferability were considered in data collection [26].

For validity in data collection [26], an experienced researcher familiar with DRSs conducted all interviews. Moreover, an acceptable time was set for data collection (from October 2019 to January 2020). Furthermore, people with different experiences and levels of activities with different perspectives were interviewed. Therefore, sampling was done from different specialties, from different cities, and different disease registries. For reliability and dependability [26], audio recordings, note-taking, and perceived nonverbal communication were used.

To increase credibility [26] in data collection, all of the interview notes were discussed in the research team meetings. In addition, 50% of the transcripts were returned to the interviewees for their approval and feedback. In addition, to improve transferability, the content of the questions, the number of participants, their characteristics, and experiences quoted by them were reported exactly.

#### Data analysis

All interviews, as well as non-verbal communication perceived by the researcher, were transcribed verbatim immediately after each interview. The transcribed texts were then entered in MAXQDA software and analyzed using Granheim and Lindgren's inductive and deductive approaches of content analysis method [27]. To minimize the researchers' bias, both authors impartially and separately identified the meaning units and openly coded the phrases and paragraphs. In the next step, to reduce the number of codes and their convergence, they identified the codes that were conceptually related and similar and classified them under different categories, and also identified the subthemes. Then, by comparing the subthemes and identifying their relationship, the themes emerged. The researchers discussed them by comparing their analyses and agreed on conflicts during the weekly discussions. Selected quotations of themes and sub-themes are also provided to complete the findings (Additional file 1: Tables S1, S2). We reported 32 Consolidated criteria for Reporting Qualitative studies (COREQ) [28] in the Additional file 2: Table S1).

In data analysis, three rigor criteria including (1) reliability, (2) credibility, and (3) confirmability was considered. To increase the reliability of data analysis [26], two researchers transcribed the interviews separately but in parallel, and matched their transcripts with the audio files in a joint session. Then, they separately coded and analyzed the interviews anonymously and finally compared their analyses. Furthermore, the codes were reviewed by a third researcher outside the research team.

For credibility [26], all of the interview notes were discussed in the research team meetings. The confirmability [26] of the data was also obtained with the final agreement of the two researchers on the classifications obtained from the analyses of the interviews. Also, after analyzing the findings, a quantitative study was performed for triangulation; the results of which confirm the findings of the qualitative section.

### Phase 2: Prioritizing identified barriers and facilitators

#### Study participants

Sampling was performed purposefully. The inclusion criteria were having at least two years of experience as a principal investigator or executive director of DRSs. 20 candidates, including the interviewees, were invited, and finally, 15 people participated.

#### Data collection

A questionnaire with closed questions was developed to prioritize the factors identified in the previous phase. This questionnaire consisted of 157 questions (63 barriers, 76 facilitators). The questions were designed in the form of a 5-point Likert scale from 1 (no important) to 5 (very important). The content and face validity of the questionnaire were approved by five DRS specialists. The reliability of the questionnaire was determined by calculating the Cronbach's alpha (a = 0.8).

#### Data analysis

The average score of each factor and theme was calculated and classified at four quartiles (25% of scores). To this end, the first quartile (less than 25% of score) was considered the fourth priority factors (mean ≤ 1.25 out of 5). We also considered the second quartile (25–50% of score) as the third priority factors (1.26 ≤ mean ≤ 2.5), the third quartile (50–75% of score) as the second priority factors (2.51 ≤ mean ≤ 3.75), and the fourth quartile (more than 75% of score) as the first priority factors (3.76 ≤ mean).

## Results

### Phase 1: Qualitative study

According to Tables 1 and 2, 8 participants were female (61.5%). The degree of the individuals was mainly clinical (n = 7, 53.8%) and their work experience was on average 8 years. Most participants were from national registries (n = 9, 69.2%).

**Fig. 1:**
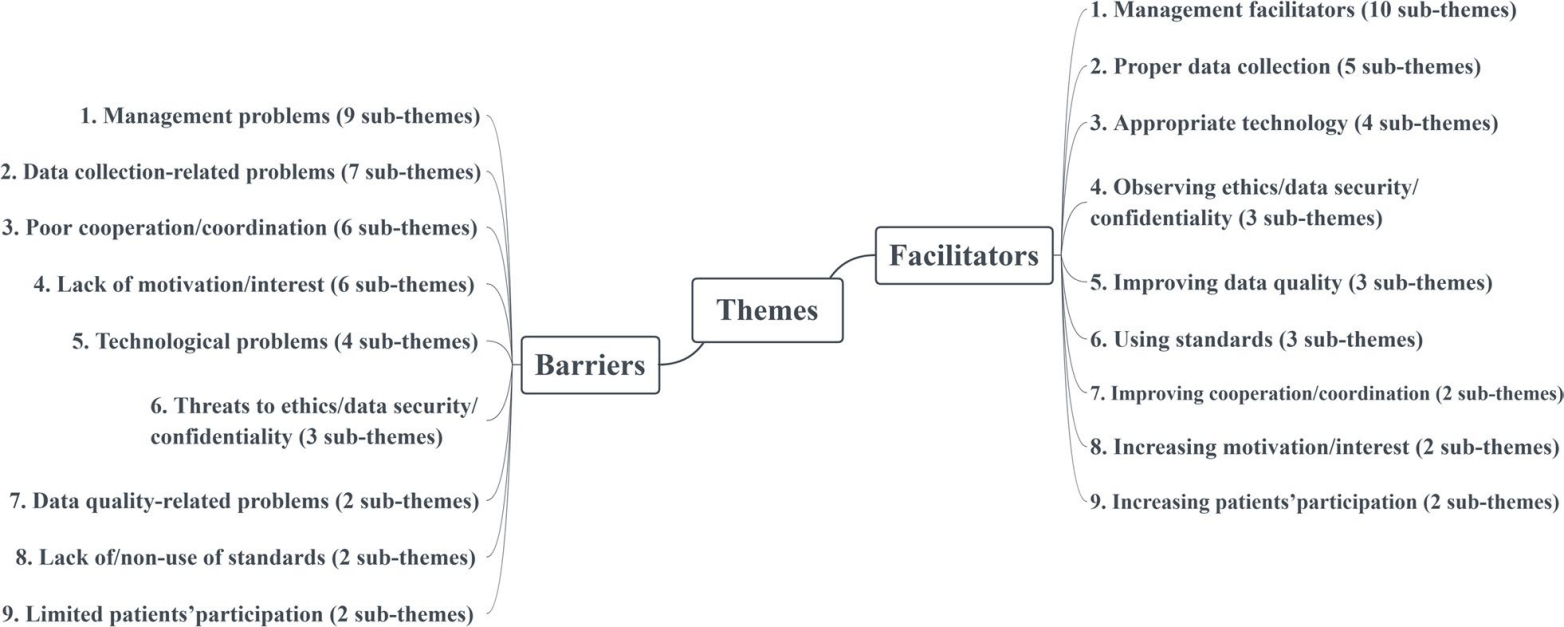
Final themes and sub-themes

We identified a total of 467 meaning units and organized them into 139 unique codes (63 barriers and 76 facilitators). We finally classified barriers-related codes in 9 themes including (1) management problems, (2) data collection-related problems, (3) poor cooperation/coordination, (4) lack of motivation/interest, (5) technological problems, (6) threats to ethics, data security, and confidentiality, (7) data quality-related problems, (8) lack of or non-use of standards and (9) limited patients’ participation. Facilitators were also classified into 9 themes including (1) management facilitators, (2) proper data collection, (3) appropriate technology, (4) observing ethics, data security, and confidentiality, (5) improving data quality, (6) using standards, (7) improving cooperation/coordination, (8) increasing motivation/interest and (9) increasing patients’ participation (Fig. 1).

### Barriers

Table 3 shows a summary of the identified barriers. Further details are in Additional file 1: Table S1.

**Table 3:** Themes and sub-themes identifying barriers for DRSs

Themes	Sub-themes 1	No of participants	Meaning units
Management problems	Resource related problems	12	40
	Organizational problems	4	10
	Insufficient awareness and education	4	7
	Steering committee-related problems	4	6
	Wrong strategic policies	4	4
	Lack of unified guidelines and protocols for standardization of DRS functions	4	7
	Rapid changes of policy makers and managers	4	6
	Problems related to registry managers	3	5
	Problems with purposes formulation	2	3
Data collection-related problems	Case-finding related problems	3	5
	Restrictions of retrospective data collection from paper records	3	3
	High volume of data elements defined for DRSs	2	2
	Incompleteness of data in hospital information systems as a data source	1	1
	Failure to comply with the data collection guidelines in DRSs	1	1
	Inconsistencies in data collection from different data sources	1	1
	Non-cooperation of physicians in the process of collecting data	1	1
Poor cooperation/coordination between stakeholders	Lack of coordination and cooperation of different stakeholders in a DRS	4	5
	Developing separate and parallel DRSs with different systems	4	6
	The difficulty of coordination between provincial (regional) DRSs in multicenter registries	3	5
	Limited and non-continual cooperation of physicians with DRSs	2	2
	Lack of coordination between universities and inter-sectoral cooperation	2	2
	Non-cooperation of data sources with the DRSs	2	3
Lack of motivation and interest	Mandatory entry of data into the registry system by staff while on duty	1	1
	Increasing employee workload through the registry functions	1	1
	Employees' fear of changes in the work process following the implementation of a DRS	1	1
	Lack or limitation of financial incentives	1	1
	The concern of physicians about the transparency of their performance through the registration of their patients’ data	1	2
	Lack of transparency of registry benefits for participants	1	4
Technological problems	Lack of technology support	5	8
	Restrictions on the data exchange between DRSs and other information systems	5	8
	Internet disruption and its low speed in Iran	2	2
	Non-use of user-friendly software in registries	2	3
Threats to ethics, data security and confidentiality	Data confidentiality issues	2	2
	Lack of transparency of data ownership	2	3
	Non-backup of data stored in DRSs	1	1
Data quality-related problems	Sources of data defects and errors	6	11
	Different measurement units of variables in different diagnostic and treatment centers	2	2
Lack of or non-use of standards	Not using data standardization	6	11
	Lack of other registry-related standards (such as reporting standards, functions, etc.)	1	6
Limited patients’ participation	Lack of patients’ participation for follow-up	4	11
	Non-cooperation of physicians in referring patients to the registries	1	3

#### Management problems

Budgetary and financial shortages and constraints and the increased costs such as manpower costs are some of the barriers that were repeatedly mentioned.

"We do not have a specific budget line for any type of registry at the Ministry of Health" (P9)

"Well, we have a lot of financial problems that always bother us. For example, we needed new staff that we wanted to hire and we wanted to keep them that not leave us because of wages but it has a cost for us. "(P2)

Lack of manpower and human resources, especially lack of skilled and trained staff and higher turnover are important problems:

"Staffs leave in the middle of the work. The new staffs need repeated training. They may go for any reason, for example, because they are students and leave and do not want to stay anymore, or they find a better job"(P2)

In Iran, the needs assessment for implementing the registries and the conditions/diseases for which implementing a DRS is a higher priority are not clear, and this can lead to a waste of resources:

"I saw that there were no priorities, for example, the ministry did not specify what priorities are in the field of eye diseases for registration and that the needs are not assessed and the specific budgets for this work are not planned in advance” (P7).

Some stated that DRSs do not have an appropriate structure and governance, or that DRSs are not usually individual-independent.

"The absence of organizational governance on which the registry is implemented is one of the major limitations that may be particularly threatening any registry or indeed its continuity" (P9)

"These programs are usually dependent on one person, for example, if one person goes and someone else comes instead, the DRS may stop" (P12)

Lack of a precise protocol for implementing and executing these systems causes inconsistencies in the processes of DRSs:

"Maybe I can tell you that registries are now implemented based on the experiences that registry managers gain during their work and trial and error, and there is currently no standard protocol in this field, and this has led to inconsistencies between DRSs."(P12)

According to some opinions, continuous changing of managers disrupts the execution of registries:

"Imagine, for example, once a manager or a new group comes and says no, these people cannot run this registry, then they stop the registry ..." (P1)

#### Data collection-related problems

In some DRSs, case definitions are not clearly defined and it leads to diversity in cases and related data:

"Case definition is often not done clearly. We see this diversity and confusion in most registries; they have difficulties in defining the case. (P9)

According to the participants, increasing the number of data variables in DRSs will eventually result in registry failure:

"If the data is not specified and explained correctly, then the registry may reach a situation where its work stops at all due to the very large amount of data set" (P9)

Physicians' non-cooperation in the data collection process is one of the other challenges mentioned:

"Especially in multidisciplinary registries, for complete data, we really need the cooperation of physicians not only in our field, but also physicians in other disciplines to collect data, but unfortunately they usually do not cooperate" (P6)

#### Technological problems

The most important problem in this field is the impossibility of interoperability between the DRSs and other information systems, which prevents data exchange and reuse of previously collected data:

"Registries cannot use each other data and unfortunately it is not possible to exchange and communicate. It is good that we are all linked but not" (P13)

#### Poor cooperation and coordination between stakeholders

One of the important barriers is the lack of proper coordination and cooperation between stakeholders. In addition, the lack of coordination of registration centers due to the lack of coordinating offices/staffs causes problems in multicenter registries:

"There is definitely a lack of coordination and cooperation between stakeholders in the registries, for example, when we wanted the consensus of cities (regional DRS centers) to determine the minimum data set, we had no cooperation" (P7)

"The more registry centers there are, the more this number will cause inconsistencies in data collection" (P7)

Limited communication and lack of interactive cooperation between university stakeholders and non-obligation of the centers to cooperate with DRSs are other problems that registries face:

"One of the problems we always had was the lack of cooperation between the universities; for example, in the registry implemented by one of these universities, the other universities did not cooperate"(P3)

"There is no plan to require centers to cooperate with DRSs ... not only for the private sector but also for university hospitals" (P12)

#### Lack of or non-use of standards

Participants believe that the lack of or non-use of data standards is an important factor that prevents the establishment of consistent and coordinated DRSs:

"It has not been considered to standardize the data. Everyone is working on their own data format and the software does not follow these standards "(P5)

"Next is the issue of data structure, which is usually full of problems. We do not have standard data at all" (P1)

#### Threats to ethics, data security, and confidentiality

Access to patients’ confidential information and insufficient attention to it in registries endangers the sensitive information of patients and reduce the desire to participate in DRSs:

"Sometimes it happens that patients’ personal information is compromised, for example, one of our registrars did not pay enough attention and gave a patient's personal information to a researcher, and this caused the patient complains" (P9)

The lack of transparency in the ownership of a DRS and its data is one of the barriers that reduce the participation of different groups:

"The fact is that we do not have a single policy for the intellectual property of data in the country, even if it is, its implementation is not very accurate and strong, and this is due to the lack of a single regulation in this regard "(P8)

#### Limited patients’ participation

Lack of patients’ participation in the registries due to concerns about the imposition of costs on them, is one of the barriers related to the registry activities, especially for follow-ups:

"Travel costs are important and heavy for many patients, so they do not cooperate" (P6)

### Facilitators

Table 4 shows the facilitators for DRSs. Further details are in the Additional file 1: Table S2.

**Table 4:** Themes and sub-themes identifying facilitators for DRSs

Themes	Sub-theme1	No of participants	Meaning units
Management facilitators	Appropriate resource management	13	40
	Increasing awareness and education	8	19
	Organizational facilitators	8	19
	Formation of scientific and executive teams	7	14
	Establishing registry guidelines	5	8
	Appropriate composition of the steering committee members	5	5
	Qualified managers	4	7
	Understanding the purpose of DRSs	4	5
	Evaluation of DRS	2	5
	Conducting feasibility study before implementing a DRS	1	1
Proper data collection	Exact definition of cases to be included in DRSs	6	7
	Appropriate data set (minimum data set)	5	8
	Collecting registry data from electronic health record system	4	4
	Collecting data during its generation (in the routine clinical process)	2	5
	Hiring appropriate data collectors	2	2
Using appropriate technology	Interoperability and integration of registry software with other information systems	8	10
	Providing appropriate software	4	8
	Working with successful and famous IT vendors in the field of registry software	2	2
	Proper data storage and backup	2	2
Observing ethics, data security and confidentiality	Developing legal guidelines	4	5
	Observing patients’ data confidentiality	4	8
	Developing security measures in software	2	2
Improving data quality	Monitoring and evaluating data quality	9	29
	Preventive n measures against data errors	4	4
	Continuous follow-up to complete the missing data	2	3
Using standards	Standardization of data in DRSs	4	4
	Using clinical coding (terminology) standards	1	1
	Using data exchange standards to communicate with the electronic health record systems	1	1
Improving cooperation/coordination	Cooperation and coordination between registries	4	5
	Group and team collaboration between DRS stakeholders	4	4
Increasing motivation and interest	Hiring interested people for DRSs	4	4
	Taking a variety of measures to increase interest and motivation	3	7
Increasing patients’ participation	Attempts to attract patients’ participation	7	10
	Obtaining informed consent and fully explaining the goals of patients’ participation to patients	3	3

#### Management facilitators

Experts believe that one of the most important management strategies is to finance the registry system and use of research budgets for financing, as well as planning to earn money from the results and outputs of DRSs:

"At the time of the reduction of the budgets, we used the so-called research budgets, which were in the form of research projects on registry data. In my personal opinion, this is the most stable and reliable type of budget that DRSs can use" (P9)

"One of the reasons we can get funding is that our data is used, for example, in student dissertations, so this is a benefit that the X or Y school might also benefit from. For example, we say that the dear director of University X, if you give us funding, we will support your students' dissertations in return "(P9)

Providing counseling and training services can help people who want to run or implement a DRS to carry out their work successfully:

"In my opinion, developing a registry is a science that has a framework. There is also a book for it. Now, most doctors who go to set up a registry, should know this framework" (P5)

"In quality controls, you may need intermediate training to teach employees how to register cases" (P4)

Developing stable organizational governance for DRSs can also be important in advancing these systems:

"For DRSs, an organizational position should actually be considered as an organizational chart." (P9)

Managers and policymakers should pave the way for proper disease registry implementation and prevent wasting resources by providing a roadmap and priorities:

"The best approach of the Ministry of Health is to develop a comprehensive roadmap and say that, for example, in the next 5 to 10 years, these diseases are very important for us, and to start developing national registry programs for them" (P12)

The development of good executive and scientific teams for developing DRSs is another management facilitator mentioned by experts:

"We definitely want a flexible IT team with the experience to support the registry" (P4)

"A scientific team should be formed to determine what should be included in the registry" (P9)

"It is also very important that there is an executive team that follows the functions of a registry, entering data, quality control and those things that are related to the executive work ..." (P1)

Developing appropriate guidelines result in the consistent implementation of DRSs in different centers:

"For every DRS, there should be a protocol that tells us what the inclusion and exclusion criteria are. This is very important when the number of people involved in the registry is increased so that everyone can work according to a single guideline." (P12)

One of the important facilitators is evaluating progress, or providing periodic reports of these systems to stakeholders:

"To achieve better outputs, the ministry should require each registry to report on the progress and review of its system at specified intervals, which will certainly help evaluate the registries." (P7)

A basic business plan is also an important factor in the proper maintenance of the registries and their financing:

"To develop the budget attachment from the beginning, we want a professional business plan that determines the costs in details from the beginning and offers solutions." (P4).

#### Using appropriate technology

Data exchanges between registries and other systems are crucial in optimizing the execution of DRSs and the easy data sharing:

"It is very good that we are all (DRSs) connected with each other, for example, patients may die in another city and be registered in the death registry of that city. We must be able to achieve the cause of death from that registry to use in scientific studies "(P13)

The provision of user-friendly software is important for simplifying the registration and optimization of DRS functions:

" We simplified this software and told the pathology centers that, in the end, if you want to get a monthly report of the data for yourself, just copy and paste it to the text box that is related to our registry system "(P9)

#### Observing ethics, data security, and confidentiality

Formulating appropriate rules for data access can increase the participants’ trust and increase their participation:

"If there is a standard in this field that explains the transfer of data and access to data, it is much better and all DRSs should use it collaboratively." (P12)

"We provide patients’ information to researchers without a name and a phone number and address, so that in any case this data does not reach anyone and does not cause further disturbances for the patient, even commercial disturbances" (P13)

#### Improving data quality

Factors such as continuous evaluation of the data quality, the use of evaluation indicators, and data quality controls are very effective in increasing the quality of data, and managers of DRSs should develop a specific plan for this issue:

"We require our registry to have a QQ attachment or Quality Control and Quality Assurance and according to its QQ specified in the proposal, the data should be collected in that format."(P8)

"Data quality assessment should be based on a set of indicators. For example, in the cancer registry, there is a criterion for the percentage of unknown data for age, sex, and in all variables specifically."(P9)

#### Increasing motivation and interest

Monetary or non-monetary incentives can increase the interest and motivation of DRS employees and stakeholders because it is important and valuable for them:

"Give proper payment to motivate people to pursue this troublesome task (DRSs related) and enter data accurately" (P12)

"We should do registry projects in the form of research projects, peoples will work in the form of research projects because it is very valuable to them." (P9)

#### Improving cooperation and coordination between stakeholders

Increasing compromise and agreement between DRSs is a kind of cooperation between them and there should be cooperation and executive coordination between national registration centers:

"We did something important. We made an agreement about the registry signed by the Deputy Minister of Research, the Deputy Minister of Health and the Deputy Minister of Treatment."(P1)

"To develop a national registry, first of all, you have to create coordination between the provinces and coordinate with the other registry centers." (P1)

"The Ministry should be able to establish communication and coordination between universities and research centers in the implementation of registries." (P12)

#### Using standards

Data standardization is the solution for aggregating data in different registries that can make information exchange possible:

"Information must be aggregated through data standardization so that it is possible to exchange data between systems and aggregate data in one place. Registries must be based on standard data "(P5)

#### Increasing patients’ participation with DRSs

Increasing patients’ participation can help improve their registration, especially regarding follow-up data. To this end, the goals of this participation, especially its benefits for patients, should be clear for them.

"To encourage patients to cooperate and participate with the registry, we provide many free services so that the patient thinks that this cooperation is for his/her benefit "(P6)

### Phase 2: Priorities of identified barriers and facilitators

Out of 20 invited experts, 15 people (75%) participated in the study. Table 5 shows the distribution of the participants.

**Table 5:** Distribution of the participants in phase 2

Characteristics	Phase 2 participants (n = 15)	
	Number (percent)	
Age	< 40 years	5 (33.3)
	≥ 40 years	10 (66.6)
Gender	Female	7 (46.6)
	Male	8 (53.3)
Duration of work in the field of DRS (years)	< 5	3 (20)
	Equal to and more than 5	12 (80)
Activity in the field of DRS (each participant may have more than one item)	Supervisor	8 (53.3)
	Evaluator	3 (20)
	Principal investigator	5 (33.3)
	Executive director	11 (73.3)
	Administrator	1 (6.6)
	Registrar	4 (26.6)
	Researcher	7 (46.6)
Field of study (each participant may have more than one item)	General Surgery	1 (6.6)
	Healthcare Services Management	1 (6.6)
	Medical Physiology	2 (13.3)
	Epidemiology	2 (13.3)
	Health Information Management	3 (20)
	Optometry	1 (6.6)
	Maternal and Child Health	1 (6.6)
	General Practitioner	3 (20)
	Nutrition	1 (6.6)
Type of degree	Clinical	8 (53.3)
	Non-clinical	4 (26.6)
	Both clinical and non-clinical	3 **(**20)
Geographical scope of registry	National	10 (66.6)
	Regional	5 (33.3)
Type of registry	Clinical-based	3 (20)
	Clinical/population-based	5 (33.3)
	Research/clinical-based	3 (20)
	Research/clinical/population-based	4 (26.6)

According to Table 6, except for "limited patients' participation" (mean = 3.63), all identified categories of barriers had a high priority (mean > 3.75). Among these, problems related to poor cooperation and coordination had the highest priority (mean = 4.30). Following that, lack of or non-use of standards (mean = 4.26), and data quality-related problems (mean = 4.06) were the next priorities.

**Table 6:** Participants’ mean score regarding barriers for DRSs

Category of barriers	Barriers	Mean ± SD	Mean ± SD
1.Poor cooperation/coordination between stakeholders	The difficulty of coordination between provincial DRSs in multicenter registries	4.40 ± 0.10	4.30 ± 0.12
	Limited and non-continual cooperation of physicians with DRSs	4.40 ± 0.10	
	Lack of coordination between universities and inter-sectoral cooperation	4.40 ± 0.10	
	Developing separate and parallel DRSs with different systems	4.33 ± 0.03	
	The reluctance of medical centers to cooperate with people and out-of-center DRSs		
	4.33 ± 0.03		
	Lack of coordination and cooperation of different stakeholders in a DRS	4.13 ± 0.17	
	Non-obligation for medical centers to cooperate with DRSs and provide data	4.13 ± 0.17	
2. Lack of or non-use of standards	Not using data standardization	4.26 ± 0	4.26 ± 0
	Lack of other registry standards such as reporting standards, functions, etc	4.26 ± 0	
3. Data quality-related problems	Different measurement units of variables in different diagnostic and treatment centers	4.26 ± 0.20	4.06 ± 0.17
	Missing data due to lack of past information or follow-up of patients	4.00 ± 0.06	
	Human errors in entering data into DRS	3.93 ± 0.13	
4.Data collection-related problems	Non-cooperation of physicians in the process of collecting data	4.33 ± 0.29	4.04 ± 0.15
	Inconsistencies in data collection from different data sources	4.13 ± 0.09	
	Incompleteness of data in hospital information systems as a data source	4.13 ± 0.09	
	Unclear definition of case (inclusion and exclusion criteria)	4.00 ± 0.04	
	Failure to comply with the data collection guideline	4.00 ± 0.04	
	Restrictions of retrospective data collection from paper records	4.00 ± 0.04	
	The disagreement of stakeholders on identifying and defining cases	3.86 ± 0.18	
	High volume of data elements defined for DRSs	3.86 ± 0.18	
5.Lack of motivation and interest	Lack of transparency of registry benefits for participants	4.06 ± 0.11	3.95 ± 0.30
	Lack or limitation of financial incentives	4.46 ± 0.51	
	The concern of physicians about the transparency of their performance through the registration of their patients’ data	4.00 ± 0.05	
	Increased employee workload due to the registry functions	3.86 ± 0.09	
	Employees' fear of changes in the work process following the implementation of DRS	3.73 ± 0.22	
	Mandatory entry of data into the registry system by staff while on duty	3.60 ± 0.35	
6. Threats to ethics, data security and confidentiality	Lack of specific data ownership regulations	4.20 ± 0.28	3.92 ± 0.30
	Non-backup of data stored in DRSs	4.20 ± 0.28	
	Lack of data confidentiality and security standards in data sharing	4.00 ± 0.08	
	Researchers' access to patients' personal and identity information	3.60 ± 0.32	
	Unauthorized access to confidential and sensitive patients’ information	3.60 ± 0.32	
7.Management problems	Lack of needs assessment by ministry of health and universities to implementing DRSs	4.40 ± 0.50	3.90 ± 0.29
	Lack of skilled and trained staff	4.26 ± 0.36	
	Manpower costs	4.20 ± 0.30	
	Lack of unified guideline and protocol for standardization of DRS functions	4.20 ± 0.30	
	Non-allocation of resources according to the priorities and necessities of the DRS in Iran	4.06 ± 0.16	
	Lack of long-term planning of DRSs by ministry of health and universities	4.06 ± 0.16	
	The dependence of DRSs on individuals (not on systems)	4.06 ± 0.16	
	Rapid changes of policy makers and managers	4.06 ± 0.16	
	Instability of staff in DRSs	4.06 ± 0.16	
	Insufficient knowledge of how to implement DRSs	4.00 ± 0.10	
	Unstable organizational structure and an appropriate steering committee for DRSs	4.00 ± 0.10	
	Implementing a DRS without having clients to use its results	4.00 ± 0.10	
	Implementing DRSs only for the purpose of using individual benefits	4.00 ± 0.10	
	Lack of specific budget for DRSs	3.93 ± 0.03	
	Server cost		3.93 ± 0.03
	Lack of connection of a DRS to an essential health service	3.86 ± 0.04	
	Lack of evaluation of DRSs by ministry of health and universities	3.86 ± 0.04	
	Cost of equipment, software and hardware	3.80 ± 0.10	
	Not identifying the scope of DRSs by managers and investigators	3.80 ± 0.10	
	Non-applicability of some DRS purposes	3.66 ± 0.24	
	Lack of participation of various specialists in steering committees	3.60 ± 0.30	
	Managers' desire to implement a DRS because it is a mode	3.40 ± 0.50	
	Lack of continuous training workshops for DRSs	3.33 ± 0.57	
	Lack of familiarity of applicants for implementing DRSs with clinical and medical sciences	3.20 ± 0.70	
8.Technological problems	Restrictions on the data exchange between DRSs and other information systems	4.46 ± 0.63	3.83 ± 0.38
	Lack of support of universities for providing servers for DRSs	4.06 ± 0.23	
	Limited technical support for the DRS by the ministry of health	3.86 ± 0.03	
	Lack of appropriate maintenance and IT support by IT vendors	3.73 ± 0.10	
	Internet disruption and its low speed in Iran	3.53 ± 0.30	
	Lack of user-friendly software used in registries	3.40 ± 0.43	
9.Limited patients’ participation	Non-cooperation of physicians for referring patients to the registries	3.93 ± 0.30	3.63 ± 0.42
	Lack of patients’ participation for follow-up	3.33 ± 0.30	

Table 7 shows that all identified facilitator categories have a high priority (mean > 3.75). But among these, improving the quality of data had the highest priority (mean = 4.54), followed by increasing motivation and interest (mean = 4.48), observing ethics, data security, and confidentiality (mean = 4.36) issues, proper data collection, and using standards (mean = 4.28, for each), respectively.

**Table 7:** Participants’ mean score regarding facilitators for DRSs

Category of facilitators	Facilitators	Mean ± SD	Mean ± SD
1.Improving data quality	Continuous evaluation of the data quality	4.80 ± 0.26	4.54 ± 0.23
	Using data quality indicators to evaluate DRSs	4.73 ± 0.19	
	Continuous follow-up to complete the missing data	4.66 ± 0.12	
	Verification and auditing of data collected from patients	4.66 ± 0.12	
	Using data prevention controls	4.60 ± 0.06	
	Presence of a data quality auditor	4.46 ± 0.08	
	Homogenization of measurement units	4.26 ± 0.28	
	Feedback on data quality to DRS employees	4.13 ± 0.41	
2.Increasing motivation and interest	Giving research motivations to employees	4.53 ± 0.05	4.48 ± 0.07
	Hiring interested people for DRSs	4.53 ± 0.05	
	Creating financial or non-financial incentives to increase people's interest in registry	4.40 ± 0.08	
3.Observing ethics, data security and confidentiality	Development of common and clear standards and guidelines for access to DRS data	4.60 ± 0.24	4.36 ± 0.16
	Developing security measures in software	4.53 ± 0.17	
	Observing ethical and legal considerations related to patients in the DRS guidelines	4.40 ± 0.04	
	Non-disclosure of patients’ information without their consent	4.33 ± 0.03	
	Obtaining consent from patients to use his/her information	4.33 ± 0.03	
	The anonymity of reports and outputs of DRSs	4.20 ± 0.16	
	Development of the intellectual rights and data ownership regulations	4.13 ± 0.23	
4.Proper data collection	Determining the appropriate and uniform minimum data set	4.80 ± 0.52	4.28 ± 0.24
	Exact definition of cases to be included in DRSs	4.53 ± 0.25	
	Informing data collectors/abstractors about the purpose of the data collection	4.40 ± 0.12	
	Omitting unnecessary data items from the defined data set	4.33 ± 0.05	
	Collecting data during its generation (in the routine clinical process)	4.20 ± 0.08	
	Collecting registry data from electronic health record system	3.93 ± 0.35	
	Data collection by physicians	3.80 ± 0.48	
5. Using standards	Using of clinical coding (terminology) standards	4.53 ± 0.25	4.28 ± 0.26
	Standardization of data in DRSs	4.33 ± 0.05	
	Using data exchange standards to communicate with the electronic health record systems	4.00 ± 0.28	
6. Improving cooperation/coordination	Coordination of provincial DRS centers with the national registry program (central office)	4.60 ± 0.40	4.20 ± 0.48
	Group and team collaboration between DRS stakeholders	4.33 ± 0.13	
	Collaboration between similar DRSs	3.66 ± 0.54	
7.Using appropriate technology	Appropriate software support by the IT company or technical team	4.40 ± 0.30	4.10 ± 0.17
	Working with successful and famous IT vendors in the field of registry software	4.13 ± 0.03	
	Using a single server for multicenter DRSs	4.13 ± 0.03	
	Interoperability and integration of registry software with other information systems	4.06 ± 0.04	
	User-friendly registry software	4.00 ± 0.10	
	Using a single, central server to store data	3.86 ± 0.24	
8.Management facilitators	Continuous evaluation of DRSs	4.60 ± 0.55	4.05 ± 0.33
	Increasing the awareness and skills of human resources in performing DRS-related tasks	4.40 ± 0.35	
	Strong scientific and executive team for the DRS	4.40 ± 0.35	
	Formulating accurate and transparent purposes for DRSs	4.40 ± 0.35	
	Developing periodic reports to evaluate the progress of DRSs	4.40 ± 0.35	
	Efforts to maintain staffs (for example, by proposing a research plan or raising wages)	4.33 ± 0.28	
	Advising and educating stakeholders in the field of DRS	4.33 ± 0.28	
	Data quality control training for employees	4.33 ± 0.28	
	Establishment of a registry secretariat in all partner universities/participants in a DRS	4.33 ± 0.28	
	Hiring managers with strong social relationships and the ability for consensus-building	4.33 ± 0.28	
	Conducting feasibility study before implementing a DRS	4.26 ± 0.21	
	Developing a specific organizational charts and structures for DRSs	4.20 ± 0.15	
	Developing a multidisciplinary team to lead DRSs	4.20 ± 0.15	
	Hiring managers with the appropriate background and practical experience in setting up DRSs	4.20 ± 0.15	
	Consensus-building of a team of experts to initiate an DRS	4.13 ± 0.08	
	Knowledge of the principal investigator in the field of the disease/condition that is going to be registered	4.13 ± 0.08	
	Determining the needs and priorities for implementing DRSs	4.13 ± 0.08	
	Using research project funding to fund DRSs	4.13 ± 0.08	
	Using ministry of health budgets for financing DRSs	4.06 ± 0.01	
	National meetings to transfer and share knowledge and experiences	4.06 ± 0.01	
	Implementing DRSs in organizations with sustainable structure and governance (such as research centers)	4.06 ± 0.01	
	Using scientific and updated guidelines and standards	4.06 ± 0.01	
	Planning to make money from DRS	4.06 ± 0.01	
	Reducing various costs such as using free, open source software, etc	3.93 ± 0.12	
	Creating an appropriate IT team to provide technical support for DRS	3.93 ± 0.12	
	Developing and upgrading the protocols for DRSs	3.93 ± 0.12	
	Developing a single, unique executive protocol at the ministry of health for all DRSs	3.93 ± 0.12	
	Presence of a representative of the involved participants and stakeholders in the management team of a DRS	3.93 ± 0.12	
	Efforts to hire staffs from various sources (such as student research centers)	3.86 ± 0.19	
	Increasing the reputation and credibility of the DRS (for example, gaining the support and approvals of the ministry of health)	3.86 ± 0.19	
	Using international guidelines as a model for developing registry protocols	3.80 ± 0.25	
	Personal financial independence in DRSs	3.73 ± 0.32	
	Connecting DRSs to the necessary clinical care and service	3.66 ± 0.39	
	Using provisional, training staff as a workforce	3.33 ± 0.72	
	Presence of the patients' representative in the meetings of the registry management committee	3.20 ± 0.85	
	Membership of a representative from all universities in the national disease registry committee in the ministry of health	3.20 ± 0.85	
9.Increasing patients’ participation	Obtaining informed consent and fully explaining the goals of patients’ participation to patients	4.06 ± 0.15	3.91 ± 0.11
	Considering therapeutic benefits and patient care	3.86 ± 0.05	
	Paying the costs of patients' cooperation with DRSs from the registry budgets	3.86 ± 0.05	

## Discussion

Our results indicate that the barriers and facilitators for DRSs include nine final themes: (1) Poor/improved cooperation and coordination between stakeholders, (2) Lack of or non-use of/using of standards, (3) Factors related to data quality, (4). Problem related to/proper data collection, (5) Lack of/increasing motivation and interest, (6) Threats to/observing ethics, data security, and confidentiality, (7) Management factors, (8) Technology-related factors and (9) Limited/increased patients' participation. The results of the quantitative study also fully confirm the findings of the quantitative study and show that the majority of identified barriers and facilitators have high priority and importance in the Iranian healthcare system. The most important of these factors are discussed below.

### Cooperation and coordination between stakeholders

Poor cooperation and coordination between stakeholders and the lack of communication between different universities involved in DRSs result in administrative inconsistencies, parallel and separate registries, and consequently waste of resources, additional costs, and registry project failure. Pop et al. [29] and Stanimirovic et al. [14] also pointed out the lack of stakeholders’ participation and communication. Furthermore, Azadmanjir et al. [30] pointed out the weak relationships between stakeholders. One of the solutions to solve these barriers is to try to establish collaborative networks and cooperation of stakeholders at the national level as well as between provincial registry centers and national DRSs. It is recommended that registry managers are committed to attracting extensive cooperation for the implementation and continuation of DRSs.

### Standards

Lack of or non-using standards is one of the major barriers for DRSs. This problem may result in inconsistencies and differences in DRSs. Similar to our study, Bommakanti et al. [17] indicate the lack of attention to the registry standards; Surodina et al. [15] highlight the lack of awareness of data and disease registry process standards. Zullig et al. [16] also mention the lack of standardization of data collection. Strategies such as adhering to the same principles in implementing and running different DRSs, coordinated use of data standards such as clinical coding, terminologies, and data exchange standards are of great advantage. Therefore, it is recommended that DRS managers and policymakers use or develop these standards to play an effective role in the success of DRSs.

### Data quality

One of the important problems is the existence of missing data, especially for patient history or follow-up, human errors in entering data, and different measurement units from different sources, which reduce the quality of data and in hence reduce the effectiveness of DRSs. These barriers were the third-ranked important barriers emphasized by experts. Azadmanjir et al. [30] point out data deficiencies. Surodina et al. [15] emphasize poor data quality and lack of relevant and reliable data in DRSs. Mandavia et al. [23] have also noted incomplete data, poor data management, and uncertainty of data quality. Bommakanti et al. [17] also indicate the poor quality of data due to underreporting and inaccurate data in DRSs.

To prevent data errors, harmonization of measurement units, as well as identification and correction of root causes of poor data quality, should be continuously considered. Evaluation of DRS data quality should be based on specific indicators to ensure completeness, timeliness, validity and comparability of registry outputs [31, 32]. Indicators such as the percentage of missing data and speed of data collection, processing, and reporting reliable and complete data to evaluate the timeliness of the registry should be considered. Furthermore, registries should monitor the ratio of registered patients to their actual number of patients to evaluate converge of the registry. Using standard coding and classification systems along with the standard definitions for data items in accordance with internationally agreed guidelines should also be considered for comparability [2]. Therefore, it is recommended that registry managers should have a data quality plan and continuously monitor data quality and provide feedback to improve the quality of DRSs.

### Data collection

Data collection is directly related to case-finding and minimum data set. Unspecified case-finding and lack of precise definition of cases lead to the inclusion of incompatible patients. Improper selection of the minimum data set can also increase the volume of data or ignore important data. Similar factors were found by Louis et al. [9]. Pop et al. [29] also noted inappropriate patient inclusion criteria and an undefined minimum data set. Azadmanjir et al. [30] highlighted a large number of data elements as a barrier. Mandavia et al. [23] also noted disagreement over the data set. The findings of all these studies are similar to our results. Therefore, in addition to the exact definition of cases and inclusion (exclusion) criteria, and the appropriate and uniform minimum data set, other facilitators including attracting the cooperation of physicians in data collection and justifying and clarifying the purposes of using data among data collectors are recommended. In addition, to ensure accurate data collection, the data is better to be collected during clinical routines.

### Incentives and motivational factors

Lack of financial incentives and concern about the transparency of individual performance are among the factors that cause the loss of participants’ desire and motivation for cooperation. In this regard, Mandavia et al. [10] mentioned similar findings such as lack of motivation for voluntary reporting. Rakhorst et al. [33] pointed to the rejection of the DRS due to the lack of motivation in patients and healthcare providers. Financial or non-financial motivators such as research scores and participation in publications can be fundamental strategies to increase the motivation of individuals and stakeholders. In this regard, managers are advised not to ignore the interest and motivation of employees and strengthen it, and to include the implementation of various motivational strategies in their management plan.

### Ethics, data security, and confidentiality

Ethical factors, security, confidentiality, and data ownership are important in the development of DRSs. Lack of specific standards or regulations in these areas may fail DRSs because it causes unauthorized access to information, ambiguity in data ownership, and information security threats. Consistent with our findings, Pop et al. [29] pointed out the lack of data protection guidelines and the ambiguity of data ownership. Gao et al. [34] also indicated raised ethical concerns about linking registered data with other data sources. Andrew et al. [35] and Keats et al. [19] highlighted the necessity of and need for ethical approvals for DRSs and Mandavia et al. [23] indicated the inappropriate determination of information and security governance laws, access to data, and failure to specify data ownership. Clear data access guidelines prevent the disclosure of unauthorized information and maintain the confidentiality of information. Considering ethical and legal issues in the DRS guidelines to reduce concerns and disputes regarding patients' participation or use of data is highly recommended.

### Management factors

Resource management, organizational factors, and developing guidelines and policies can result in the success or failure of DRSs. These factors are very diverse but some of them have a high priority. Barriers such as increased costs, especially the cost of manpower and financial restrictions can lead to challenges such as permanent shortages and lack of stability of the staff, especially professional and skilled staff. Other studies [9, 23, 29, 30] reported similar findings such as lack of financial support and lack of manpower. Strategies such as developing a business plan, using research budgets, and involving staff in research projects can help managers reduce these problems.

One of the main reasons for the failure of DRSs is the lack of initial needs assessment, which has not been mentioned in previous studies. Without needs assessment and not paying attention to the priorities for the development of DRSs, these systems will face failure or inefficiency. Therefore, conducting a needs assessment and determining the required priorities is one of the necessities in launching DRSs. In this regard, the ministry of health, public health agencies, and university policymakers are advised to evaluate the existing needs and necessities before launching DRSs and to distribute resources based on these necessities.

The lack of specific protocols and guidelines for DRSs causes inconsistencies and differences in conducting the functions of DRSs. This may lead to other problems such as threats to the integrity of these systems. Other researchers [15, 17] highlighted the non-compliance with the protocols and guidelines in DRSs, especially in multi-center DRSs.

The dependence of DRSs on individuals causes changes in a DRS and sometimes its stops due to the elimination of that individual. This finding has not been reported in previous studies. Therefore, managers and decision-makers in the field of DRS are recommended to employ a strong and coordinated steering committee for the management of these systems instead of dependency on one person and his/her decisions.

### Technology

Technology-related problems such as the limitation for data exchanges between disease registry programs and other software systems prevent integrating with other systems and hence results in separate and heterogeneous software. In this regard, Behera et al. [36] pointed out the lack of links between DRSs. Other researchers [14, 15] highlighted the lack of interoperability, integration, and heterogeneity of information systems and DRS software. Additionally, strong IT support and user-friendliness of registry software should be considered.

### Patients’ participation

Patients' non-participation in follow-up or registry-related studies leads to inaccuracies and incompleteness in post-discharge data and disruption of these studies. Korngut et al. [37] pointed out the non-participation of patients in the registry due to concerns about the possibility of additional visits as well as transportation and financial costs associated with these visits, which is in line with our findings. Considering the benefits of patients, and accepting the costs imposed on patients to cooperate and participate are suggested.

### Study limitation and strengths

In the second phase of this study, out of 20 invited and qualified experts, five did not participate in prioritizing barriers and facilitators of DRSs. Therefore, the results of this part of the study cannot be generalized to all registries in the country. In addition, the results may not be generalizable to the registries of other countries. On the other hand, the strengths of this study were the cooperation of participants from different registries, with different roles, and from different cities across the country.

## Conclusion

The most important barriers in DRSs are problems related to stakeholders’ cooperation and coordination, non-use of standards, and problems related to data quality, which more significantly create challenges for DRSs. On the contrary, the most important facilitators and solutions are the efforts to increase the quality of data, increase the motivation and interest of stakeholders and observe ethical and legal regulations, maintain the security and confidentiality of data. Therefore, managers and policymakers of DRSs with paying more attention to the mentioned barriers and facilitators can increase the probability of success of DRSs.

## Supplementary Information

**Additional file 1.** Interview guide and sample interviewees’ quotes.

**Additional file 2.** Consolidated criteria for reporting qualitative studies (COREQ) checklist.

## Data Availability

Data sharing is not applicable to this article as no datasets were generated or analyzed during the current study.

## Abbreviations

COREQ: Consolidated criteria for reporting qualitative studies
DRS: Disease registry system
IT: Information technology
MAXQDA: MAX qualitative data analysis
QQ: Quality control and quality assurance
