# Supplementary material for: Barriers and facilitators for disease registry systems: a mixed-method study
Source: BMC Med Inform Decis Mak. 2022 Apr 11;22:97. doi: 10.1186/s12911-022-01840-7 (PMC9004114; doi:10.1186/s12911-022-01840-7)
Supplement: Supplementary file 1 — Additional file 1. Interview guide and sample interviewees’ quotes. [file 12911_2022_1840_MOESM1_ESM.docx]

Additional file 1

Interview guide

Table 1- Sample interviewees’ quotes related to barriers

Table 2- Sample interviewees’ quotes related to facilitators

Interview guide

1. What is your experience with disease registry systems (registrar, researcher, supervisor, executive manager, etc.)?)?

2. Can you describe the details of your experience?

3- What issues and problems have you encountered in your work related to disease registry systems so far?

4. What were your solutions to encounter these issues and problems?

5. Can you explain some examples of the problems and the solutions?

6. Apart from the above, what other issues and problems do you think may affect disease registry systems in the country?

7- What is your suggested solution to encounter these issues and problems?

8-How do you think these facilitators and solutions may help to disease registry systems in the country?

Table 1- Sample interviewees’ quotes realted to barriers

| Themes | Sub-theme level 1 | Sub-theme level 2 | Sample Quotes |
| --- | --- | --- | --- |
| Management problems | Resource related problems | Lack of specific budget for DRSs | "Unfortunately, the registries do not have the necessary allocation for financial assignments in the ministry." (P10) |
|  |  | Manpower costs | "Other significant costs were the salaries and expenses of the registrars that we had to pay, which were really heavy." (P5) |
|  |  | Cost of equipment, software and hardware | "Another big cost is hardware costs and registry software costs." (P12) |
|  |  | Server cost | "The next issue is hardware. We have to store our information, so we have to buy a server, but the problem is that the server cost is high." (P12) |
|  |  | Lack of skilled and trained staff | "When you have not professional staff in the field of the registry or staff aware of the data, so you do not have scientific support. In this case, the wrong and missing data will be increased." (P10) |
|  |  | Instability of staff in DRSs | "Another problem was that the staff were not permanent and worked with us on a part-time basis." (P11) |
|  | Organizational problems | Lack of needs assessment by ministry of health and universities to implementing DRSs | "One of the biggest problems is the lack of needs assessment for registry implementation in the ministry." (P7) |
|  |  | Non-allocation of resources according to the priorities and necessities of the DRS in Iran | "Resource allocation for registries is not based on priorities." (P10) |
|  |  | Lack of evaluation of DRSs by ministry of health and universities | "The third problem is the lack of a formal evaluation of the registries. I can boldly tell you that no evaluation is done on the registries by the disease registry unit in the ministry." (P7) |
|  |  | Lack of long-term planning of DRSs by ministry of health and universities | "Until now, there has been no long-term vision and planning for registries, how these registries should be managed in the country." (P8) |
|  | Insufficient awareness and education | Insufficient knowledge of how to implement DRSs | "Most people who recommend a registry implementation are not familiar with its dimensions and do not really know what to do to implement a registry." (P8) |
|  |  | Lack of continuous training workshops for DRSs | "Previously, the Ministry held a training course about DRSs but stopped it. The non-continuation of these training courses was one of the mistakes or problems that exists." (P13) |
|  | Steering committee-related problems | Unstable organizational structure and an appropriate steering committee for DRSs | "We have more than 160 registries in the country that have at least their titles confirmed. But how many of these systems are active now? This is because the organizational structure of these registries is not well developed and there is no specific structure for them at all." (P5) |
|  |  | Lack of participation of various specialists in steering committees | "We did not have experts before the implementation of a registry, that is, at the DRS model development stage. I mean, there are, but we had limitations in this area, we did not have many experts to be able to use their information ".(P7) |
|  | Problems related to registry managers | Lack of familiarity of applicants for implementing DRSs with clinical and medical sciences | "A researcher may have an idea to set up a registry, but this person is neither clinical nor has he/she worked in medical centers. Well, can this person set up a disease registry?" (P3) |
|  |  | Not identifying the scope of DRSs by managers and investigators | "Many of registry applicants do not really know the depth of the registry program, that is, they do not identify the scope of work and do not think about how much effort, time or cost it takes." (P3) |
|  |  | Managers' desire to implement a DRS because it is a mode | "Like the cohort atmosphere in the country where many people like to run a cohort without any need, when several registries are set up, a registry implementation atmosphere is created, which means that sometimes the launch of these registries is common and everyone like to do this."(P13) |
|  | Wrong strategic policies | Implementing a DRS without having clients to use its results | "If we do not have a customer for the registry, that is, the customer who needs our data, we can never justify the problems and create a solution for them." (P9) |
|  |  | The dependence of DRSs on individuals (not on systems) | "for example, a specialist )a doctor) likes to do some research work, he launches a registry, then, for example, if he is not in the project for a while ... that project stops, meaning that only this registry depends on his work and his presence or absence." (P5) |
|  | Lack of unified guideline and protocol for standardization of DRS functions | | "Now there is no such thing if I want to operate in the registry based on a guideline and follow a specific protocol." (P4) |
|  | Rapid changes of policy makers and managers | | "After two or three years in some universities, if a manager changes, the new director who comes believes that a registrar who has worked for three years and four years and is experienced is suitable for another job." (P9) |
|  | Problems with purposes formulation | Implementing DRSs only for the purpose of using individual benefits | "Many people want to implement a DRS because of the privileges that universities have for implementing a registry." (P5) |
|  |  | Lack of connection of a DRS to an essential health service | "Why when a child is born, his family has to go and register him in the Civil Registration? In order to be able to get a birth certificate for the child. Now the same should be done for our registry, who depends on our registry? Who will be deprived of such an important service if it is not registered in our system? Nobody." (P8) |
|  |  | Non-applicability of some DRS purposes | "For example, we have a hospital infection registry that has practically no use. What does it mean? That is, if in this system it is determined that the nosocomial infection is caused by the performance of a doctor, there should be a reaction to the performance of him, but what? Nothing is done." (P8) |
| Data collection-related problems | Case-finding related problems | Unclear definition of case (inclusion and exclusion criteria) | "Most cases that are probable, suspicious, definite, etc. are not precisely defined and can not be distinguished from each other, and people in the registry often have problems in these cases." (P8) |
|  |  | The disagreement of stakeholders on identifying and defining cases | "Registry stakeholders did not agree on the cases, for example, some people said why not collect benign cases or why such cases should not be included?" (P9) |
|  | High volume of data elements defined for DRSs | | "Sometimes the number of variables and the amount of data that had to be collected was so large that our registrars did not have time to collect and the data remained incomplete." (P8) |
|  | Restrictions of retrospective data collection from paper records | | "If you want to review patient records, you have to retrospectively extract information from these files. This information is not very classified and it takes a long time to produce it.Therefore, this is difficult to do and the data may be collected incorrectly."(P13) |
|  | Incompleteness of data in hospital information systems as a data source | | "To collect registry data items, everyone gave a lot of comments for example they said we should include these items as HIS data items, but the problem is that this data item may not be filled like other items that are not usually filled in the HIS." (P5) |
|  | Failure to comply with the data collection guideline | | "One of the biggest challenges in our registry was that even though we had a guideline to collect data, it was not followed by everyone." (P7) |
|  | Inconsistencies in data collection from different data sources | | "Regarding the registry, it should be said that because the data sources are in different places, that is, the data is in different sources, there are certainly some inconsistencies in the collection of this data." (P9) |
|  | Non-cooperation of physicians in the process of collecting data | | "Doctors are an important member of the registry team, but they usually do not cooperate in completing the files properly and their files are incomplete and out of line." (P2) |
| Poor cooperation/ coordination between stakeholders | Lack of coordination and cooperation of different stakeholders in a DRS | | "There is definitely a lack of coordination and cooperation of stakeholders in the registries, for example, when we wanted the consensus of cities (DRS regional centers) to determine the minimum data set, we had no cooperation." (P7) |
|  | Developing separate and parallel DRSs with different systems | | "Now each university or research center sets up its own registry and has no cooperation with any registry else." (P12) |
|  | The difficulty of coordination between provincial (regional) DRSs in multicenter registries | | "When you want a national registry, it becomes difficult because first of all, you have to create coordination between the provinces, and creating this coordination is also difficult and can be one of the big problems." (P1) |
|  | Limited and non-continual cooperation of physicians with DRSs | | "The cooperation of doctors in our registry was limited, they cooperated on a part-time basis. For example, when the information from the registry was given to them, they did not give feedback on the data due to their busy schedule." (P11) |
|  | Lack of coordination between universities and inter-sectoral cooperation | | "Practically, interactions between departments such as different universities and different research centers in the implementation of a registry are not as coherent as they should be." (P12) |
|  | Non-cooperation of data sources with the DRSs | Non-obligation for medical centers to cooperate with DRSs and provide data | "There is no plan to require centers to cooperate with DRSs, not only for the private sector but also for university hospitals." (P12) |
|  |  | The reluctance of medical centers to cooperate with people and out-of-center DRSs | "Another challenge is that centers usually prefer that outsiders do not enter their system to collect data and usually do not cooperate with a registry." (P9) |
| Technological problems | Restrictions on the data exchange between DRSs and other information systems | | "Registries are not able to exchange information with other systems. For example, there is a lot of patient laboratory information in laboratories, but it is impossible to send this information to the registry system." (P12) |
|  | Lack of technology support | Lack of support of universities for providing servers for DRSs | "One of the problems that is very costly in the current costs of a registry is providing a server for registry information, which is very important and unfortunately universities do not consider very much." (P7) |
|  |  | Lack of appropriate maintenance and IT support by IT vendors | "You may get a registry software company and then see that next year you can not work with that company because it has no support and does not support you." (P1) |
|  |  | Limited technical support for the DRS by the ministry of health | "We need special technical support for registry. The ministry, which is responsible for all registries, should be justified in this regard and provide us with this support, but it is usually weak." (P10) |
|  | Internet disruption and its low speed in Iran | | "Our registry is web-based so the Internet should be very fast but most of the time we encounter Internet speed disturbance." (P6) |
|  | Non use of user-friendly software in registries | | "Our disease registry software was not very user-friendly." (P12) |
| Lack of motivation and interest | Mandatory entry of data into the registry system by staff while on duty | | "Some registries force people to enter the necessary information in the registry ... When there is a compulsion, employees are no longer motivated to work." (P12) |
|  | Increased employee workload due to the registry functions | | "Those who wanted to collect data resisted so as not to overburden their work and were not interested in the registry." (P9) |
|  | Employees' fear of changes in the work process following the implementation of DRS | | "One of the problems in all innovations, such as implementing a registry, is people's fear of change. Even if this change is in favor of improvement, people are worried and afraid of changes." (P8) |
|  | Lack or limitation of financial incentives | | "For example, if we can not keep employees satisfied financially, they will not find an incentive to registr cases." (P4) |
|  | The concern of physicians about the transparency of their performance through the registration of their patients’ data | | "Some people are worried about the transparency of operations. What does it mean? It means, for example, that a doctor knows that if there is a registry, it turns out that Dr. X's patient with appendicitis has been hospitalized for two days, while my patient has been hospitalized for three days. So they may put me aside and keep that doctor." (P12) |
|  | Lack of transparency of registry benefits for participants | | "If the beneficiary or the registry administrator has a negative view of the registry, that system will certainly run into problems after a while because usually, these people do not know exactly what their benefit is from the registry. Usually, their benefits are not clear from the registry." (P9) |
| Threats to ethics, data security and confidentiality | Data confidentiality issues | Researchers' access to patients' personal and identity information | "Sometimes it happens that the personal information of the registered patients is endangered, for example, in the same X-record, it happened that the personal information was given to a researcher and this caused the patient to complain." (P9) |
|  |  | Unauthorized access to confidential and sensitive patients’ information | "It has happened that the names of the registered patients have been accessed by one of the pharmaceutical companies or other unauthorized persons." (P8) |
|  | Lack of transparency of data ownership | Lack of data confidentiality and security standards in data sharing | "We generally do not have a specific standard for determining the confidentiality and security of data." (P12) |
|  |  | Lack of specific data ownership regulations | "There is no regulation to indicate how registries data is owned." (P8) |
|  | Non-backup of data stored in DRSs | | "Once our data was lost. A period of data we entered was deleted. We did not make a backup unfortunately and all data of this period was lost and could not be recovered." (P2) |
| Data quality-related problems | Sources of data defects and errors | Missing data due to lack of past information or follow-up of patients | "Most of our problems are in the incompleteness of the data we need. Usually, our patient information is not complete, such as previous information or patient follow-up information." (P6) |
|  |  | Human errors in entering data into DRS | "The user may not be careful in entering the data and may be the source of error, for example, not entering the patient's weight. It may be important that the weight has increased by half a kilo, but the accuracy of the recording was one kilo." (P4) |
|  | Different measurement units of variables in different diagnostic and treatment centers | | "For example, the test units were not the same or the tests were recorded without units because our patients went to different laboratories." (P13) |
| Lack of or non-use of standards | Not using data standardization | | "When standard data is not used, everyone can fill in data from their own points of view." (p4) |
|  | Lack of other registry-related standards (such as reporting standards, functions, etc.) | | "The necessary standards have not been specified for the registries. For example, it does not explain exactly what tasks are in the registries." (P12) |
| Limited patients’ participation | Lack of patients’ participation for follow-up | | "When we ask a patient a research-related data, patients may not cooperate because they may feel they are under study." (P13) |
|  | Non-cooperation of physicians for referring patients to the registries | | "Another problem is the lack of cooperation of physicians in referring patients to the registry program. They always refer patients from different places for the first time, but then do not refer again." (P6) |

DRS: Disease Registry System

HIS: Hospital Information System

IT: Information Technology

Table 2- Sample interviewees’ quotes realted to facilitators

| Themes | Sub-theme level 1 | Sub-theme level 2 | Sample Quotes |
| --- | --- | --- | --- |
| Management facilitators | Appropriate resource management | Personal financial independence in DRSs | "We hired people with our own budget and personal resources to do the registry work for us, and we were able to pay the employees." (P10) |
|  |  | Planning to make money from DRSs | "Each cell of the registry data tables has a specific monetary value that must be planned before launching the registry." (P4) |
|  |  | Using ministry of health budgets for financing DRSs | "We used the budget from the ministry because our registry was national." (P5) |
|  |  | Using research project funding to fund DRSs | "One way to receive funds is to request from academic centers to fund the registry in the form of separate research projects." (P7) |
|  |  | Reducing various costs such as using free, open source software, etc. | "Using ready-made software helps a lot in terms of reducing costs because once it was programmed, it means that once this design cost was paid and now it is used for everyone. Thus, the cost of buying software is very low." (P5) |
|  |  | Increasing the awareness and skills of human resources in performing DRS-related tasks | "People who worked for the registry should be trained, from registrars to nurses and staff who cooperate with the registry." (P6) |
|  |  | Using provisional, training staff as a workforce | "Now, because many universities do not fund recruitment, they request for training staff to enter information in the registries." (P12) |
|  |  | Efforts to hire staffs from various sources (such as student research centers) | "For registries whose registrars must be clinicians, our solution is to cooperate with the student research centers of medical universities, for example, students who like to do research will be employed in the registry. This provides a CV for students and provides a way for their research work." (P5) |
|  |  | Efforts to maintain staffs (for example, by proposing a research plan or raising wages) | "We suggest to the staff and students who are working in the registry to carry out a research project based on our data so that they can stay because of their projects." (P2) |
|  | Increasing awareness and education | Advising and educating stakeholders in the field of DRS | "We have to educate researchers or those who are interested in the registry about the registry methodology and framework. The registry is not an executive discussion, it has a methodology that should be known and taught." (P5) |
|  |  | Data quality control training for employees | "However, once in a while we usually hold a workshop to discuss the quality control of the registry." (P1) |
|  |  | National meetings to transfer and share knowledge and experiences | "We held a workshop in the field of registry. We held international conferences and meetings and invited everyone to come and share their information with others." (P3) |
|  | Organizational facilitators | Determining the needs and priorities for implementing DRSs | "Prioritization helps a lot to develop a good registry that we really need, such as a registry for a disease that has a higher burden on society in terms of health outcomes."(P7) |
|  |  | Increasing the reputation and credibility of the DRS (for example, gaining the support and approvals of the ministry of health) | "As soon as the registry program seeks the support of the ministry, this support itself is a credit and facilitator for that registry." (P3) |
|  |  | Establishment of a registry secretariat in all partner universities/participants in a DRS | "We created a virtually X registry secretariat in all universities with two to three registrars so that the university could have a good national registry." (P9) |
|  |  | Developing specific organizational charts and structures for DRSs | "We have to design a structure for the registry to say that we want to move forward with this structure ... That is, we want to determine if this structure is better? Or that? Or, for example, whose should we hire?" (P1) |
|  |  | Implementing DRS in organizations with sustainable structure and governance (such as research centers) | "A registry should be established in places such as research centers or in places that are so-called stable in terms of manpower and structure." (P9) |
|  | Formation of scientific and executive teams | Creating an appropriate IT team to provide technical support for DRS | "We had the chance to have a well-thought-out and experienced IT team at the service of our registry team that we were able to use their knowledge and create very good registry outputs." (P13) |
|  |  | Strong scientific and executive team for the DRS | "After making decisions in the steering committee, there must be a team that executes the decisions and has the necessary expertise and knowledge to execute the registry." (P1) |
|  | Establishing registry guidelines | Developing and upgrading the protocols for DRSs | "There should be a standard guideline and protocol for each disease registry separately so that the creation, implementation, continuation, and evaluation of registries is not a matter of taste and is based on a specific guideline." (P7) |
|  |  | Using scientific and updated guidelines and standards | "There should be a group that scientifically supports the registry because in creating the registry, scientific and updated disease guidelines should be used." (P1) |
|  |  | Developing a single, unique executive protocol at the ministry of health for all DRSs | "There should be a standard protocol by a registry executive body, such as Ministry at the macro level, so that everyone can develop their registry based on the standard protocol." (P7) |
|  |  | Using international guidelines as a model for developing registry protocols | "A special format of guidelines (according to international standards) should be provided and made available to registrars." (P11) |
|  | Understanding the purpose of DRSs | Formulating accurate and transparent purposes for DRSs | "To create a specific registry protocol, first you have to write the purpose of that registry. It has to be very clear, that is, you have to know exactly what you want." (P7) |
|  |  | Connecting DRSs to the necessary clinical care and service | "What do they say in the TB registry? They say we only give TB medicine to those who are registered in the registry with this regulation, so if the registry is connected to a service and care, such as a drug distribution service, no manager can stop it. Do you understand?" (P8) |
|  | Appropriate composition of the steering committee members | Presence of the patients' representative in the meetings of the registry management committee | "One of the good things has happened to us, inspired by international articles and registries, was that we also had a patiets’ representative at the registry steering committee." (P7) |
|  |  | Membership of a representative from all universities in the national disease registry committee in the ministry of health | "The Ministry should include all representatives and officials of the university registry programs in its disease registry committee to use the potential of all universities in policy-making." (P12) |
|  |  | Presence of a representative of the involved participants and stakeholders in the management team of a DRS | "In the management team of the registry, many people, from the director of IT, the director of medical records to the head of the departments participate. They should be in this council to say what each of them did or can do for the registry." (P1) |
|  |  | Developing a multidisciplinary team to lead DRSs | "We should be looking for a comprehensive team in the registry. This team should be multidisciplinary, ie an IT expert, an expert to follow the details of the work, a person or group of people specializing in the disease, registrars, and one or more advisers for researchers to use the registry must be on this team." (P4) |
|  |  | Consensus-building of a team of experts to initiate an DRS | "We tell DRS applicants that in order to be able to create a successful registry, you must form a steering committee based on the consensus of experts in the field." (P3) |
|  | Conducting feasibility study before implementing a DRS | | "The researcher who wants to set up a registry must do the feasibility study. Who can launch this program? Can he collect his data or not? Is there a financial resource and facilities for this work? Can that scientific group be formed?" (P3) |
|  | Qualified managers | Knowledge of the principal investigator in the field of the disease/condition that is going to be registered | "The applicant researcher must be aware of what he or she wants to do and the registry he or she wants to set up." (P3) |
|  |  | Hiring managers with strong social relationships and the ability for consensus-building | "Registry managers must have strong social relationships, be able to communicate with people, be able to persuade employees to stay and help them." (P4) |
|  |  | Hiring managers with the appropriate background and practical experience in setting up DRSs | "The fact is that the registry manager must have some practical experience in this field (registry) and just with reading a book can not launch the registry." (P8) |
|  | Evaluation of DRSs | Developing periodic reports to evaluate the progress of DRSs | "To achieve better outputs, the ministry should require each registry to report the progress and review of its system at specified intervals, which will certainly be helpful in evaluating the registries." (P7) |
|  |  | Continuous evaluation of DRSs | "Registry evaluation should be done continuously and at any level, ie at the level of presenting a model of a registry, at the level of launching the registry, in different phases of its implementation, whether national, regional, or hospital phases." (P7) |
| Improving data quality | Monitoring and evaluating data quality | Feedback on data quality to DRS employees | "Percentage of different data errors are reported both in the whole registry and for each data item and feedback is given to registry users." (P9) |
|  |  | Continuous evaluation of the data quality | "In our registry, the quality of the data is monitored as much as possible and is checked continuously and periodically." (P11) |
|  |  | Using data quality indicators to evaluate DRSs | "Evaluation of registry data quality should be done based on a series of criteria. For example, in our registry, one of the criteria for data quality is the percentage of unknown values for each variable separately." (P4) |
|  |  | Presence of a data quality auditor | "We have a quality control officer, which is very important because he randomly pulls out some of the recorded items and matches the patient records to see how accurate the data is." (P2) |
|  |  | Verification and auditing of data collected from patients | "To control the quality and accuracy of the data, you extract the information from a sample of the registered patients or their records and match it to the registry data to see if it is really being recorded correctly or not." (P4) |
|  | Preventive measures against data errors | Using data prevention controls | "One of the mechanisms of quality control of registry data is preventive control so that we can apply preventive controls by educating users or designing default data values to prevent typing errors." (P12) |
|  |  | Homogenization of measurement units | "Over time, we realized that we need to make corrections in the unit of variables and have specific units." (P13) |
|  | Continuous follow-up to complete the missing data | | "Sometimes in data sources, some data is not filled and the reported cases are incomplete. Then we say or call and follow up to correct the data." (P2 ) |
| Proper data collection | Exact definition of cases to be included in DRSs | | "The definition of the disease and the patient case of that registry must be specified. What is the method of identifying cases? All these cases must be defined." (P7) |
|  | Appropriate data set (minimum data set) | Determining the appropriate and uniform minimum data set | "The registry they want to design and the variables they have in mind should be appropriate to the registry from the beginning, for example, what data they want or what data they do not want." (P6) |
|  |  | Omitting unnecessary data items from the defined data set | "We have to define that questionnaire and the DRS minimum data set correctly and remove the additional and unnecessary information." (P9) |
|  | Collecting registry data from electronic health record system | | "For registries, it is easy to find all patients who have referred to all centers by searching for the disease code in EHR database. For example, demographic information, medications taken and patient history." (P12) |
|  | Collecting data during its generation (in the routine clinical process) | | "The correct way to collect data is that when the data is generated, it must be entered to the registry." (P8) |
|  | Hiring appropriate data collectors | Informing data collectors/abstractors about the purpose of the data collection | "Explain to those who collect the data what the use of the information they are collecting is, what its purpose is." (P9) |
|  |  | Data collection by physicians | "Now in our registry, the doctor himself is collecting the data, which helps a lot to collect the data accurately and correctly." (P10) |
| Observing ethics, data security and confidentiality | Developing legal guidelines | Development of common and clear standards and guidelines for access to DRS data | "We have designed a common data access instruction for Registry X, for example, we have created a transparent process for how people can access registry data. This should be in all registries." (P9) |
|  |  | Observing ethical and legal considerations related to patients in the DRS guidelines | "We tried to include the ethical and legal considerations of our patients in the registry guideline so that these facts are taken into account in the registry." (P4) |
|  |  | Development of the intellectual rights and data ownership regulations | "We have created a set of principles for intellectual property rights in the registry to observe the three aspects of privacy, ethics, and law in the registry. For example, in addition to the patient who must have privacy, the same must be observed for the doctors who work with us." (P4) |
|  | Developing security measures in software | | "We provided software data security to protect the registry data. The data access level was determined by the ethics committee, and technically it was done by IT experts." (P8) |
|  | Observing patients’ data confidentiality | The anonymity of reports and outputs of DRSs | "We develop the registry output reports for researchers without the patient name and contact number." (P4) |
|  |  | Non-disclosure of patients’ information without their consent | "I must know that I can not disclose any information without the patient's permission or the relevant authorities and provide the patient's information to anyone." (P13) |
|  |  | Obtaining consent from patients to use his/her information | "We obtain consent from the patient to provide him or her information to various people requesting data for research." (P13) |
| Using appropriate technology | Interoperability and integration of registry software with other information systems | | "If we can integrate this registry system with the data source system from which we receive data, it will certainly make data registration much easier." (P9) |
|  | Providing appropriate software | User-friendly registry software | "Registry software should be as simple and user-friendly as possible, easy to work with, useful and easy reporting." (P1) |
|  |  | Appropriate software support by the IT company or technical team | "Registry software support from the company or IT team should be OK." (P1) |
|  | Working with successful and famous IT vendors in the field of registry software | | "It's great to work with a company that designs strong registry software." (P6) |
|  | Proper data storage and backup | Using a single, central server to store data | "To integrate registry information, it is best to have a single, integrated server in the ministry that creates a single database." (P11) |
|  |  | Using a single server for multicenter DRSs | "We wanted the various registry centers to be connected to a single server and all the data to be integrated so that the reporting and security of the server would be OK." (P2) |
| Improving cooperation/coordination between stakeholders | Cooperation and coordination between registries | Collaboration between similar DRSs | "If there were cooperation and coordination between the registries, these separate and small systems could become a national system." (P10) |
|  |  | Coordination of provincial DRS centers with the national registry program (central office) | "To create a national registry, first of all, you have to create coordination between the provincial registries and coordinate with participant centers." (P1) |
|  | Group and team collaboration between DRS stakeholders | | "To design the registry, we regularly held various meetings with all members of the steering committee and decided that it was not a one-on-one discussion and that all aspects were considered in a group." (P5) |
| Using standards | Standardization of data in DRSs | | "In creating a registry, the data must be consistent and standardized, and all information that comes from different sources must be entered to the registry in the same format and standard." (P1) |
|  | Using clinical coding (terminology ) standards | | " Coding standards should be used in registries to exchange information with electronic health records." (P12) |
|  | Using data exchange standards to communicate with the electronic health record systems | | "Given that we have an electronic health record policy, and definitely registry systems should follow this policy; information exchange standards should be used and registries should be designed to be able to connect to other systems such as EHRs." (P12) |
| Increasing motivation and interest | Taking a variety of measures to increase interest and motivation | Creating financial or non-financial incentives to increase people's interest in registry | "In order to create interest, a wage and additional payment should be considered for data entry for individuals." (P4) |
|  |  | Giving research motivations to employees | "Research suggestions motivate users to do registry-related tasks more accurately." (P2) |
|  | Hiring interested people for DRSs | | "We should try to invite new people to the registry that be more interested in doing so." (P4) |
| Increasing patients’ participation | Attempts to attract patients’ participation | Considering therapeutic benefits and patient care | "In our registry, we try to ask patients more treatment-related questions than the the research questions so that patients feel they are being asked for his or her own treatment and for his or her own profit." (P13) |
|  |  | Paying the costs of patients' cooperation with DRSs from the registry budgets | "To follow up patients in the registry, if a patient needs to be retested, you (the registry team) will have to pay for the tests for your patient so that the patient is encouraged to come." (P12) |
|  | Obtaining informed consent and fully explaining the goals of patients’ participation to patients | | "To get patients to cooperate with the registry, we first justified them and based on the informed consent we received from them, only included patients who tended to donate blood." (P11) |

CV: Curriculum Vitae

DRS: Disease Registry System

EHR: Electronic Health Record

HIS: Hospital Information System

IT: Information Technology

TB: Tuberculosis
